# Reversible Surface Energy Storage in Molecular-Scale Porous Materials

**DOI:** 10.3390/molecules29030664

**Published:** 2024-01-31

**Authors:** Dusan Bratko

**Affiliations:** Department of Chemistry, Virginia Commonwealth University, Richmond, VA 23221, USA; dbratko@vcu.edu

**Keywords:** molecular porosity, interfacial energy, wetting/dewetting hysteresis, open ensemble molecular simulations

## Abstract

Forcible wetting of hydrophobic pores represents a viable method for energy storage in the form of interfacial energy. The energy used to fill the pores can be recovered as pressure–volume work upon decompression. For efficient recovery, the expulsion pressure should not be significantly lower than the pressure required for infiltration. Hysteresis of the wetting/drying cycle associated with the kinetic barrier to liquid expulsion results in energy dissipation and reduced storage efficiency. In the present work, we use open ensemble (Grand Canonical) Monte Carlo simulations to study the improvement of energy recovery with decreasing diameters of planar pores. Near-complete reversibility is achieved at pore widths barely accommodating a monolayer of the liquid, thus minimizing the area of the liquid/gas interface during the cavitation process. At the same time, these conditions lead to a steep increase in the infiltration pressure required to overcome steric wall/water repulsion in a tight confinement and a considerable reduction in the translational entropy of confined molecules. In principle, similar effects can be expected when increasing the size of the liquid particles without altering the absorbent porosity. While the latter approach is easier to follow in laboratory work, we discuss the advantages of reducing the pore diameter, which reduces the cycling hysteresis while *simultaneously* improving the stored-energy density in the material.

## 1. Introduction

Energy absorption in the form of interfacial free energy of wetted lyophobic materials underlies the function of liquid springs, shock absorbers, and bumper materials [1,2,3,4,5,6,7,8], listed according to their decreasing degree of energy recovery. Liquid springs are characterized by their high recovery of pressure–volume work, a necessary condition for energy storage applications. In the first approximation, stored energy can be estimated as the product of the absorbent’s wetting free energy, Δγ, and wetted area. High energy density is achievable in microporous materials [9], characterized by a large surface-to-volume ratio, e.g., zeolites [2,3,6,7,10,11,12,13,14,15,16] or metallo-organic frameworks [17] with specific areas of O(10^3^) m^2^g^−1^ or higher. A satisfactory recovery, on the other hand, requires small differences between the intrusion and expulsion pressures, *P*_in_ and *P*_ex_, respectively, where $P_{in}$~$\frac{2\Delta \gamma }{h_{eff}}\;$ is the minimal pressure leading to liquid infiltration into the pores accommodating a water slab of thickness *h*_eff_, and *P*_ex_ ≤ *P*_in_ is the pressure at which the liquid is spontaneously expelled from the material. In wide pores, *h*_eff_ is close to the separation between the pore walls *h,* measured between the surface layers of atoms on each wall. The expression *h*_eff_ ~ *h* − σ provides a better approximation in narrow pores, where the thickness of a layer of wall atoms (σ) cannot be neglected in comparison to *h*. ∆γ= −γ cosθ denotes the material’s wetting free energy, γ  is the surface tension, and θ represents the contact angle of the given liquid on a flat surface made of the absorbent material. Storage efficiency can be quantified as the ratio of recovered and stored energies, $\eta =\frac{P_{ex}}{P_{in}}\leq 1,$ with η=1 corresponding to a fully reversible filling/expulsion cycle. Experimental and modeling studies show that the infiltration process (described in terms of liquid uptake in the pores as a function of pressure) usually proceeds through equilibrium states, while the expulsion typically occurs at an external pressure *P*_ex_ below *P*_in_, as the nucleation of gaseous phase proceeds through an activation barrier Ω* [6,18,19,20,21] dominated by the surface free energy of the newly created vapor nucleus at the transition state. For common pore geometries, e.g., planar or cylindrical, the capillarity theory predicts the leading contribution to the barrier Ω*  to scale in proportion to $-\gamma h_{eff}^{2}cos\theta^{-1}$ [22,23,24]. Upon the descent to the nanoscale, a linear term proportional to $-\tau h_{eff}cos\theta^{-1}$ (where τ  denotes line tension) becomes increasingly important [7,20,24,25,26,27], suggesting slower barrier scaling [7,20,26] with *h_eff_* in tight confinements. While narrowing the pores generally lowers the barrier and alleviates the level of hysteresis, reduced accuracy of continuum approximations and limited information about line tension for various water/substrate systems inhibit quantitative predictions when *h_eff_* approaches molecular dimensions. In the present work, we employ open ensemble molecular simulations of a simple absorbent model that allows us to systematically vary the diameter of the pores. We concentrate on systems with a planar pore geometry where the hysteresis is most prominent and, further, the effect of pore width is easier to isolate as *h* can be varied without changing other properties, e.g., the curvature-dependent wettability of the walls. Previous works showed water in hydrocarbon-like pores of widths *h* above ~1.6 nm to persist in a metastable liquid state [28,29,30,31] even at vanishing bulk pressure *P*_b_. Planar pores with *h* ~1 nm were found to support delayed expulsion with considerable hysteresis. With *P*_ex_ of about one-fifth of *P*_in_, the latter system can be classified as a shock absorber. Achieving liquid spring behavior requires a further reduction in pore size. To determine the porosity supporting reversible operation, we monitored the extent of hysteresis as a function of effective pore diameter using Grand Canonical ensemble simulations. These computations revealed an increased sensitivity to pore size at molecular-scale dimensions, where steric exclusion gradually turns liquid–wall interactions repulsive irrespective of the hydrophilicity of the material. The sizes of the pores and liquid molecules can also be matched by manipulating the size of liquid particles without changing the absorbent, an approach arguably easier to implement in experiments. A possible example is provided by the improved performance of devices with concentrated electrolytes replacing neat water [2,3,9,10,11,12,13,14,15,32]. In these systems, steric exclusion effects can be amplified through the abundance of hydrated ions. Along with a possible osmotic gradient [33], these effects can facilitate solution expulsion and reduce the hysteresis of the cycle. When experimentally feasible, however, tailoring the pore diameter is an attractive option because it can combine the advantages of weakened hysteresis, higher infiltration pressure *P*_in_ (at similar wettability), *and* increased specific surface area *a*, the latter two quantities scaling approximately as the inverse of *h*. The molecular simulation results presented in the following sections showcase simultaneous improvements in *both* cycling reversibility and increased stored energy density (~*a*∆γ) for a set of absorbents as the pore diameters approach the molecular size of the liquid. Lowered activation barriers indicated by reduced hysteresis are also suggestive of accelerated expulsion kinetics, potentially correlating power density with the porosity of the absorbent.

## 2. Results and Discussion

Most of the results presented and discussed in this section concern the pressure dependence of water uptake inside a porous absorbent open to the exchange of water molecules between the pores and the bulk aqueous environment. Thermodynamically stable and metastable equilibria between the bulk and confined phases are studied by open ensemble (Grand Canonical Monte Carlo) simulations, which critically depend on the knowledge of chemical potentials imposed through pressure control in the bulk phase, as detailed in Models and Methods Section (Section 3). The following paragraph describes the initial verifications of these data.

### 2.1. Benchmarking

**Figure 1:**
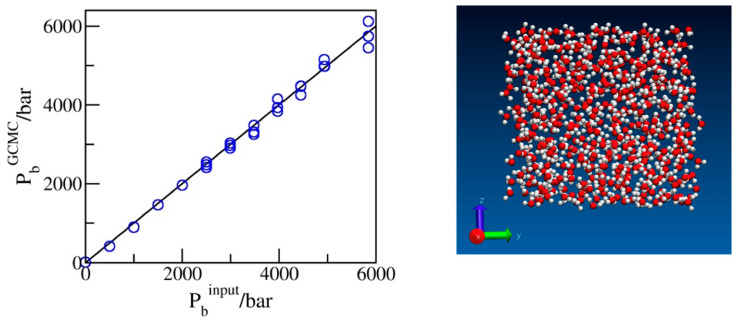
Simulated pressures of bulk water as a function of the input pressures used in predicting the excess chemical potentials for the GCMC simulations. The snapshot shows oxygen and hydrogen atoms in red and white, respectively.

Before turning our attention to confined systems, we tested the reliability of the input chemical potentials whose dependence on the bulk pressure *P*_b_ was determined from the volumetric data for water adjusted to the SPC/E model of water used in simulations (Section 3). Figure 1 presents the calculated pressures from the explicit bulk phase simulations of SPC/E water corresponding to excess chemical potentials, $\mu_{ex}\left(P_{b}\right)$, estimated as described in Section 3. The comparison of the simulated (GCMC) pressures with the input bulk pressures used in the predictions of $\mu_{ex}\left(P_{b}\right)$ (Figure 1) shows excellent agreement between the two sets of data. Slight statistical uncertainties at extreme compression are explained by the decreasing acceptance of exchange moves at these conditions. Because of the slow convergence of the average number of molecules *N* associated with small exchange acceptances, typically of O(10^−4^), runs with ~10^9^ attempted moves were typically required for satisfactory precision. An additional test of the input chemical potentials was provided by confirming that the simulated water populations (*N*) were consistent with the compressibility data for SPC/E water determined independently in isobaric simulations [34] within an estimated statistical uncertainty of ~0.5% or better. In confinement, the present scheme reproduces known results for water content in wider pores with identical water–wall interactions, obtained [31] using six-step Expanded Ensemble Grand Canonical Monte Carlo (EEGCMC), within ±1–2%. The small difference is attributed to the present use of force field-specific chemical potentials $\mu_{ex}\left(P_{b}\right)\;$ for the SPC/E model as opposed to the experimental values applied [31] in previous work.

### 2.2. Intrusion/Expulsion Cycling

**Figure 2:**
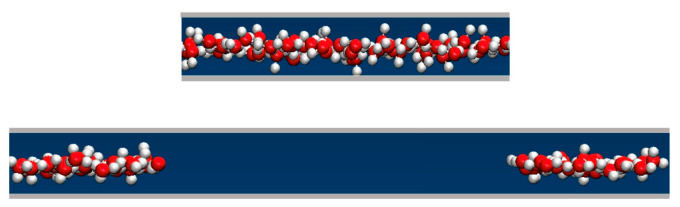
A schematic representation of a (yz) cross-section of a simulation box with a volume of L_x_ × L_y_ × h in an equilibrated, water-filled state (**top**), and the same system after the box is elongated by a factor of 2 in the Y direction. The height h is shown in the vertical direction, and the Y-axis points in the horizontal direction. The snapshot shows oxygen and hydrogen atoms in red and white, respectively (**bottom**). The cross-section of the box increased to L_x_ × 2L_y_ × h with an extra volume of L_x_ × L_y_ × h containing no water molecules. The contiguity of the original water configuration is maintained through periodic replication. Continued open ensemble simulations at P_b_ > P_in_ lead to gradual filling of the entire box, whereas the system undergoes a barrier-free evacuation if P_b_ < P_in_.

**Figure 3:**
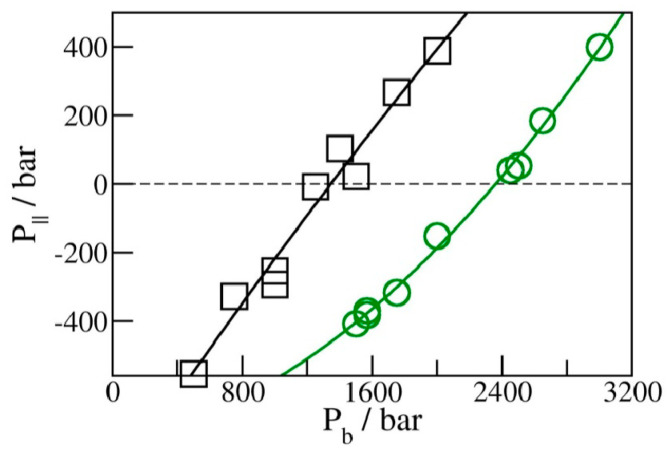
Lateral pressures P_||_ in liquid-filled planar confinements with periodic box sizes of 25 Å × 25 Å × 10 Å (black) and 25 Å × 25 Å × 8 Å (green) from GCMC simulations for the decompression branch of the filling–expulsion cycle as functions of bulk pressure P_b_. The value of P_b_ leading to vanishing P_||_ corresponds to the intrusion pressure P_in_ at a given pore width: 1.36 ± 0.04 kbar at 10 Å and 2.37 ± 0.03 kbar at 8 Å.

We determined the average water uptake in absorbent pores over a broad range of bulk pressures P_b_ from an ambient pressure of 1 bar to ~10 kbar, considering two types of initial configurations: fully wetted pores preequilibrated at a slightly higher compression, or half-full pores resembling transient configurations involved in intrusion events. Depending on the bulk pressure P_b_, water inside the nanopores in equilibrium with the bulk phase can exist in a vapor or liquid state [19,20,25,28,29,35]. The threshold intrusion pressure P_in_, which triggers an abrupt density increase from a gas- to liquid-like value, varies approximately as the inverse of the width of the confined aqueous layer. Experiments and molecular simulations performed along the compression branch tend to follow the equilibrium pressure-dependence of the fluid density in the confinement [31,36]. Conversely, on the decompression branch, a metastable liquid is commonly observed to persist in analogy to the hysteresis observed in condensation/desorption simulations [37]. Due to the activation barrier associated with the emergence of the new liquid/vapor interface during vapor nucleation, spontaneous liquid expulsion is often delayed until the bulk pressure falls significantly below the intrusion pressure P_in_. A comparable activation barrier is not involved during the liquid’s intrusion, which typically involves the propagation of a pre-existing liquid/vapor interface towards the interior of the pore [31]. During intrusion, the area of the liquid/vapor interface decreases and eventually disappears in a barrier-free process. Distinct pathways are accounted for in simulating the opposite processes of intrusion and expulsion. The points on the extrusion (decompression) branch are generated through GCMC re-equilibration in water-filled pores previously equilibrated at a somewhat higher external pressure, and the sequence of simulation runs initiated in a confined-liquid state (P_b_ > P_in_) is repeated until we reach a value of bulk pressure, P_ex_, sufficiently low to trigger the expulsion of metastable liquid from the pore. The infiltration branch corresponding to a sequence of increasing bulk pressures, on the other hand, is studied by monitoring the change in water content in runs initiated in half-filled pores we create by doubling the volume of an initially filled pore equilibrated at the desired bulk pressure P_b_ (Figure 2). This is achieved by increasing the box size by a factor of two along the Y-axis while water molecules remain in their original positions. As a result, the enlarged box contains water-filled and empty half-spaces periodically repeated in the Y direction (Figure 2). When proceeding with the GCMC simulation, the added space is gradually filled with water if P_b_ exceeds the intrusion pressure P_in_, whereas low P_b_ < P_in_ results in the withdrawal of the liquid until the entire box contains only vapor. The two situations (P_b_ above or below P_in_) are characterized by different signs of the lateral pressure inside the pore, with positive P_||_ signifying infiltration and negative P_||_ leading to expulsion [31]. The intrusion pressure, P_in_, can therefore also be defined as the value of bulk pressure P_b_ corresponding to the vanishing lateral pressure, P_||_, inside the pore. Figure 3 illustrates the determination of P_in_ by monitoring P_||_ as a function of P_b_ in water-filled systems without involving an explicit phase transition. The above example uses GCMC dependences of P_||_ on the bulk pressure on the decompression branch at pore widths h = 8 and 10 Å.

**Figure 4:**
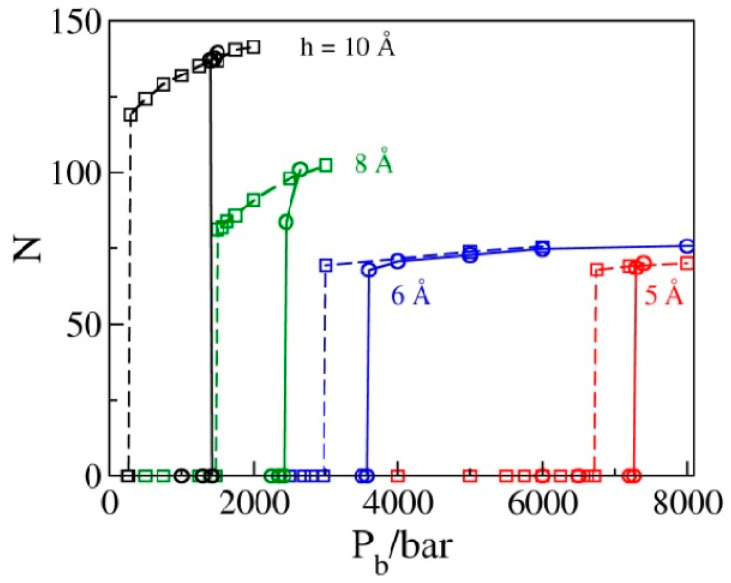
The average number of molecules corresponding to a periodic box of size 25 Å × 25 Å × h in GCMC simulations as a function of the bulk pressure P_b_ for pore widths h = 10, 8, 6, or 5 Å is determined in a sequence of runs for increasing (solid lines) and decreasing bulk pressures. Abrupt changes correspond to liquid intrusion (solid lines) and expulsion (dashed lines) events taking place at pressures P_in_ and P_ex_, respectively. Hysteresis manifested as the relative difference between intrusion and expulsion pressures, η= (P_in_ − P_ex_)/P_ex_, shows a rapid decrease with decreasing width of the pores. The intrusion branch data, obtained in boxes of size 25 Å × 50 Å × h, are prorated to the smaller box size used in the expulsion branch calculations.

Within statistical precision, the values of P_b_ corresponding to the sign change in P_||_ in liquid-filled pores on the extrusion branch equal intrusion pressures P_in_, corresponding to the water intrusion events on the compression branches in Figure 4. This figure shows the GCMC water populations N as functions of bulk pressure P_b_ in simulated pores for a set of pore diameters h = 10, 8, 6, or 5 Å determined in sequences of simulation runs at increasing pressures (compression branch) that eventually lead to the intrusion of water into the pore (solid lines), as well as sequences of states generated by gradually reducing P_b_ (decompression branch, dashed lines) until water is expelled from the pores. All results correspond to box sizes 25 Å × 25 Å × h; the numbers of molecules observed along the compression branch using bigger boxes (25 Å × 50 Å × h) are prorated to the smaller box size to allow for comparisons with the extrusion branch simulations. Abrupt changes in N correspond to liquid intrusion (solid lines) and expulsion events (dashed lines) that take place at pressures P_in_ and P_ex_, respectively. The hysteresis, manifested as the relative difference between intrusion and expulsion pressures, ξ= (P_in_ − P_ex_)/P_ex_, shows a rapid decrease with the width of the pores. Maximal energy recovery, $\eta =\frac{P_{ex}}{P_{in}}=1-\xi$, is therefore significantly improved in narrower pores.

### 2.3. Phase Transition Pressures

**Figure 5:**
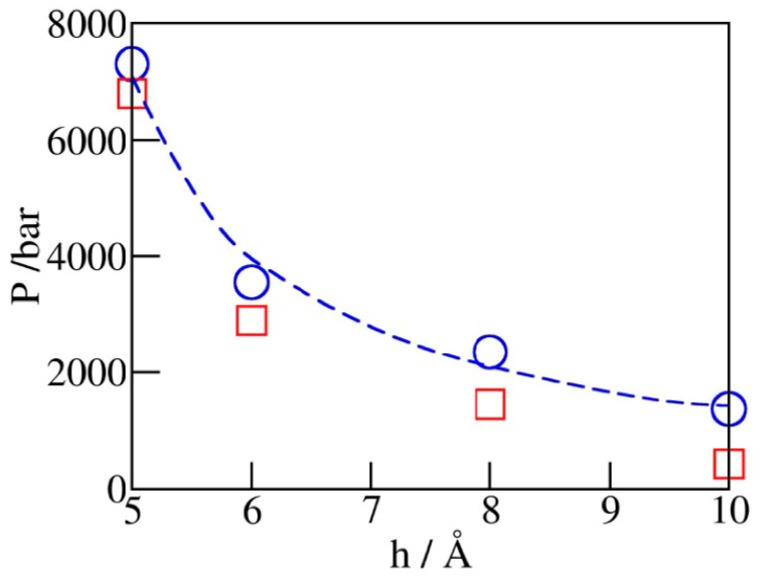
Simulated intrusion (blue circles) and extrusion (red squares) pressures as functions of pore width h from GCMC runs illustrated in Figure 4. The dashed blue line follows the continuum level prediction $P_{in}\left(h\right)≅P_{in}\left(5Å\right)\frac{5Å-\sigma_{CH_{n}}}{h-\sigma_{CH_{n}}}$, which is close to the simulation data but underestimates the slope at very small widths. The extrusion pressures (red squares) are generally lower than the intrusion ones. The relative difference, which reflects the extent of hysteresis, becomes insignificant at pore widths barely accommodating a monolayer of water.

Figure 5 shows the dependence of simulated intrusion and expulsion pressures, *P*_in_ and *P*_ex_, on the pore diameter *h*. The increased volatility of confined water observed upon the reduction of pore diameter conforms to intensified depletion of the overall hydrogen bonding and concomitant structural changes as hydration water becomes the majority component in the system [38]. The observed variation of *P*_in_ can be approximated by the macroscopic relation $P_{in}≅\frac{2∆\gamma }{h_{eff}}$, where ∆γ represents the wetting free energy of the walls and $h_{eff}\approx h-\sigma_{CH_{n}}$ is the water-accessible width of the pore obtained by subtracting the diameter of CH_n_ groups ($\sigma_{CH_{n}}=3.74\;Å\;$ for the given model) from the C-C distance (*h*) across the pore. The above relation, based on the capillarity theory and the assumption of width-independent ∆γ, captures qualitative changes in *P*_in_ but underestimates the rate of change at separations below ~6 Å, indicating a possible increase of ∆γ in this regime.

### 2.4. Liquid Structure in Narrow Pores

**Figure 6:**
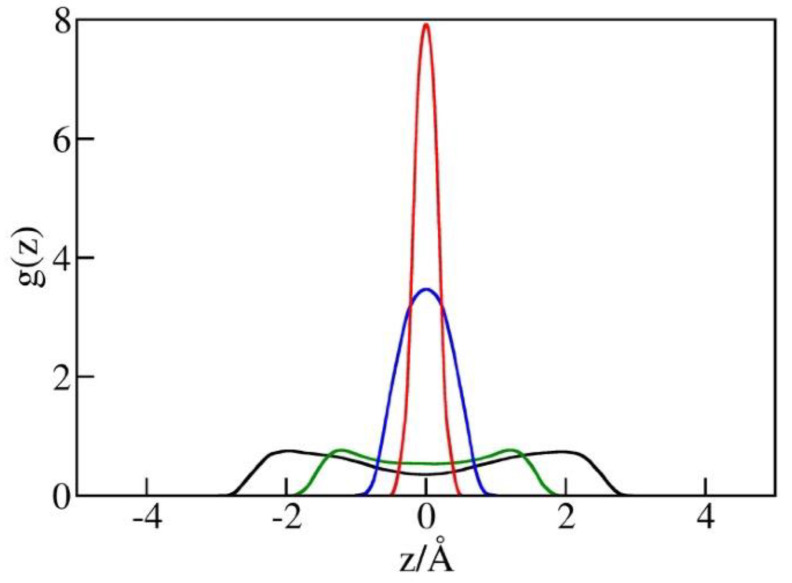
Distribution functions g(z) of water oxygen in simulation boxes of the volume 25 Å × 25 Å × h for pore diameters h = 10 Å (black), 8 Å (green), 6 Å (blue), and 5 Å (red) from GCMC simulations at bulk pressures P_b_ equal to the intrusion pressures P_in_(h) for each of the widths. The maxima of g(z) in comparatively loose pores with h = 10 Å or h = 8 Å are observed approximately 3 Å or 2.6 Å from the walls, respectively. These separations correspond to attractive water–wall interactions (Equation (1)). In narrower pores with h = 5 or 6 Å, water molecules are restricted to a narrow region close to the pore midplane, characterized by a weakly repulsive combined interaction of water molecules with both confining walls. Pore wetting at these conditions requires high external pressures (Figure 5), while easier expulsion reduces the hysteresis of intrusion/expulsion cycles, as shown in Figure 4 and Figure 5.

The possibility of increased ∆γ in the narrowest pores is supported by the observed liquid structures at different pore widths illustrated in Figure 6. This figure shows singlet distribution functions for oxygen atoms in water, *g*(z), in simulation boxes with a diameter *h* between 5 Å and 10 Å. g(z) is defined as the ratio of the average local density of *centers* of water oxygen atoms in the pore to their density in bulk water in equilibrium with the pore. The maxima of *g*(z) correspond to the minima of water/wall potential of mean force (PMF) controlled by direct wall–water interactions (Equation (1)) and packing effects. In wider pores (*h* = 10 Å or *h* = 8 Å), the maxima are observed at distances of about 3 Å or 2.6 Å from the walls, respectively. In narrower pores (*h* = 5 or 6 Å), the maximal distances, $\left|z-z_{w}\right|,$ between the centers of water molecules from either wall are smaller than *h*/2 (2.5 or 3 Å), resulting in a repulsive potential of the mean force of water molecules. As a result, molecules compressed into the pore tend to reside near the midplane of the confinement (Figure 6). High peak values of g(z) reflect in-plane ordering of water centers without suggesting comparable changes in (inverse) molar volume. In view of finite molecular size $\sigma_{O}≅3.17\;Å$, the actual volume occupied by water molecules extends about ½$\sigma_{O}$ or ~1.6 Å beyond the region populated by the oxygen centers. In the tight pore limit, where g(z) asymptotically approaches the form of the δ function, the physical thickness of the confined monolayer hence remains close to $\sigma_{O}\;$ and its average density is comparable to that of liquid water.

The combination of unfavorable energetics and reduced translational entropy of confined molecules destabilizes confined liquid relative to the vapor nucleus formed at the initial step toward evacuation. The lower activation barrier of the process results in weaker hysteresis and improved energy recovery in systems with narrow pores.

**Figure 7:**
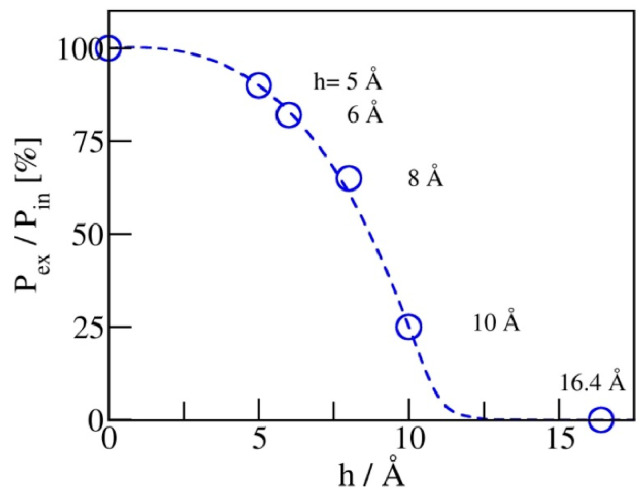
Maximal recovery of stored energy, determined by the ratio of expulsion and intrusion pressures, η= P_ex_/P_in_, approaches unity upon the narrowing of the pores. Vanishing η, on the other hand, corresponds to situations with long-lived metastable liquid persisting in the pores at arbitrarily low pressures P_b_.

The maximal recovery (before any friction dissipation [21]) can be defined as the ratio of expulsion and intrusion pressures, η= *P*_ex_/*P*_in_. Figure 7 illustrates the dependence of η on pore width. For hydrophobic pores of planar geometry, a reduction in pore width *h* from 1 nm to 0.5 nm improves the recovery from ~25 to 92%. A viable storage recovery, η≥0.75, is obtained at pore widths at or below 6 Å, where only a monolayer of water molecules can be accommodated inside the pores. Restricting the pore size to a molecular scale in at least one dimension is apparently sufficient to secure the absorbent’s liquid spring behavior.

### 2.5. Lyophilic Absorbent

**Figure 8:**
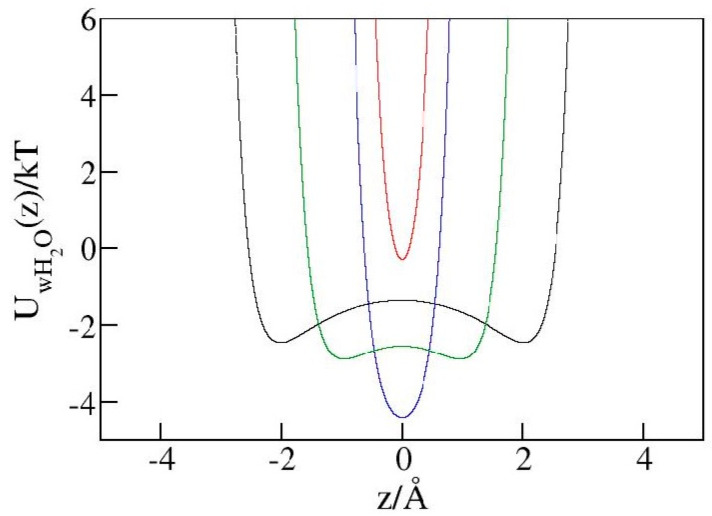
Interaction of a water molecule with both hydrophilic walls as a function of position inside the pores of widths h = 10 Å (black), 8 Å (green), 6 Å (blue), and 5 Å (red). The most favorable distance from either wall is about 2.97 Å, while the interaction turns repulsive at a wall–water distance below ~2.46 Å. At wall–wall separation h = 6 Å (blue line), the molecules can achieve optimal interactions with both walls. In the narrow 5 Å pore (red line), however, the maximal possible distance from the nearer wall is 2.5 Å, allowing only near-neutral or repulsive combined water–wall interactions.

As discussed in the context of Figure 6, steric restrictions can result in a purely repulsive water/wall interaction even when short-ranged attraction is exerted on a freely accessible surface made of identical wall material. Analogous reasoning suggests the enhancement of steric effects at molecular porosity could potentially lead to hydrophobic-like behavior in nanopores, irrespective of the macroscopic hydrophilicity of the absorbent material. To explore this possibility, we performed GCMC simulations in hydrophilic pores with diameters of *h* = 5 Å or 6 Å using identical geometries as in the calculation in hydrophobic pores. To render the walls nominally hydrophilic, we used a stronger water/wall LJ potential (Equation (1)) with the wall atom LJ energy parameter $\varepsilon_{w}$ (previously 0.648 kJ mol^−1^) increased to 5 kJ mol^−1^. This change corresponds to an increase of mixed wall/water $\varepsilon_{LJ\;}$ from 0.63 kJ mol^−1^ to 1.8 kJ mol^−1^, ambient wall/water contact angle reduction from ~127° to ~84.5 ± 2°, and ambient wetting free energy (∆γ) reduction from (positive) 38 dyn cm^−1^ to (negative) −6 dyn cm^−1^. Consistent with the negative sign of ∆γ, GCMC simulations for pore widths *h* ≥ 6 Å showed water retention (η = 0) at any bulk pressure, meaning that water intrudes under all conditions and changes in bulk pressure cannot trigger expulsion. At the smallest pore width, *h* =5 Å, where steric repulsion dominates water/wall interaction, however, the system manifests hydrophobic behavior with an intrusion pressure *P*_in_ of ~7.5 kbar. The reason for this qualitative change is explained in Figure 8, which shows this tight confinement does not provide sufficient space for water molecules to reside at energetically favorable distances of ~3 Å from the walls. The use of more detailed atomistic models of confining walls can shift precise positions of wall–water contact distances and interaction minima but should preserve the qualitative picture conveyed in Figure 8. In a study of pore size effects in a related problem of gas condensation in lyophilic adsorbents, Deroche et al. identified a “reminiscent capillarity regime”, where a uniform scaling with effective pore width (corresponding to *h*_eff_ or *h* − σ in our present notation) was obeyed as long as the adsorbate molecules could comfortably fit into the pores [39]. According to Figure 8, the latter condition is no longer fulfilled in our narrowest-pore system. Lacking wall–water attraction, this system cannot retain water upon decompression. Expulsion is consistently observed at *P*_ex_~6.38 kbar. At the given pore width (5 Å), the hydrophilic material hence supports a storage capacity comparable to the hydrophobic one. At about 85%, the energy recovery is only 9% lower than in the hydrophobic absorbent. Further narrowing of the pores is likely to reduce the remaining differences between the two materials, making molecular-scale porosity the main determinant of the absorbent’s usefulness in liquid springs for energy storage applications. This said, their better overall performance supports the use of hydrophobic materials in pragmatic contexts. Fundamental aspects of confinement-induced lyophobic transition invite broader and more systematic investigations that exceed the scope of the present study and are deferred to future work.

### 2.6. Limiting Energy Density

Using a set of simplifying assumptions, we can estimate the available energy per unit mass (or volume), $\bar{u}$, for the water-filled model absorbent. To account for the contribution of the pore walls to the total mass, we assume a realistic thickness of pore walls $\delta_{w}$ of ~1 nm and the rounded density of the entire system ρ of ~1 g cm^−3^. The energy absorbed during intrusion can be approximated as *P*_in_∆V and the work recovered during expulsion as *P*_ex_∆V, where $∆V=∆N\bar{V}\;$ equals the product of the number of absorbed or expelled molecules times the partial molecular volume ($\bar{V}$) in bulk water at a respective (intrusion or expulsion) pressure. Useful work *P*_ex_∆V is associated with the mass *m* = ρ*L*_x_*L*_y_(*h* + $\delta_{w}$). Using ∆N values from Figure 4, we obtain the upper limits of accessible energy densities $\bar{\;u}$ during the hypothetical friction-less operation at 14 J g^−1^, 30 J g^−1^, 63 J g^−1^, and 147 J g^−1^ for pore widths *h* of 10 Å, 8 Å, 6 Å, and 5 Å, respectively (see the Graphical Abstract). The literature estimates of dissipation losses in zeolite absorbents were reported [16] in the range of 2–3 J g^−1^. The rapid increase in $\bar{u}$ with decreasing *h* reflects a number of factors, including the increases in *P*_ex_ and the specific area density (wall area per unit mass), as well as the remarkably weak dependence of ∆N on the pore width (*N* = 80, 75, and 69 ± 2 at widths *h* = 8, 6, and 5 Å, respectively) in narrow pores barely accommodating a single monolayer of the liquid. The above energy densities between 14 and 147 J g^−1^ compare reasonably well to the available experimental data [17] for zeolite/electrolyte systems with high end cases for recoverable energy storage between 20 and 30 J g^−1^ and the highest experimental energy absorption as high as 93 J g^−1^ [8,40]. The molecular simulations described above suggest further improvements are possible by carefully tuning the pore dimensions or by pairing the available absorbents with solvents of a matching molecular size. Interestingly, focusing on conditions with strong steric effects results in a weaker sensitivity to the remaining characteristics of surface/solvent interactions, including the material’s liophobicity on macroscopic surfaces.

### 2.7. Polydispersity

So far, we have solely been concerned about monodisperse porous materials, where all pores are of a planar geometry with identical diameters. Experiments are, however, often performed using materials with discrete or continuous distributions of pore geometries. In contrast to monodisperse samples, this feature leads to multi-step liquid uptake and expulsion processes or continuous transitions with finite slopes of intrusion and expulsion branches constituting the wetting/dewetting cycle. In view of weak correlations [41,42,43] between intrusion/expulsion events in distinct pores, results for a spectrum of pore sizes should enable predictions for water content in a polydisperse absorbent as a linear superposition of fractional contributions from pores of different sizes. In a general case, a normalized pore size distribution function *p*(*h*), where *p*(*h*)d*h* represents the fraction of pores of widths between *h* and *h* + d*h*, determines the volume change of the bulk phase associated with the absorption of *N*($P_{b})$ molecules in the material at pressure *P*_b_, ∆*V(P*_b_*)* = $-\bar{V}\left(P_{b}\right)N\left(P_{b}\right),$ where $\;\bar{V}\left(P_{b}\right)$ is the partial molar volume in bulk water at pressure *P*_b_, $N\left(P_{b}\right)=\int_{0}^{\infty }N\left(P_{b},h\right)p(h)dh,$ and *N*$\left(P_{b},h\right)$ denotes the average number of absorbed molecules inside a pore of width *h* at bulk pressure *P*_b._ To consider practically relevant systems like zeolites, which are often characterized by a discrete distribution over a few pore geometries, it is necessary to combine simulation results analogous to those illustrated in Figure 4 for representative pore shapes while replacing the above integral with a discrete summation. Interpolations between the simulated $N\left(P_{b},h\right)$ data may be necessary to describe a polydisperse absorbent with a continuous pore-type distribution. Because of their extreme sensitivity to pore size, the hydrophilic materials discussed in Section 2.5 can only perform well in their monodispersed form. In view of their better tolerance with respect to pore width distribution, hydrophobic absorbents remain strongly preferred as surface energy storage materials.

## 3. Models and Methods

### 3.1. Models

We modeled porous absorbents as systems composed of planar pores consisting of parallel hydrocarbon-like walls separated by distance *h.* We considered systems with *h* = 5 Å, 6 Å, 8 Å, or 10 Å. The separation was measured between the planes corresponding to the presumed positions of carbon atoms in water-exposed CH_n_ groups on opposite walls. Separations above 1 nm were not considered, as even the use of a 1 nm width resulted in prohibitive hysteresis for pragmatic purposes. Notably, ~1.5 nm and wider pores have been shown to retain absorbed water [31] in a metastable liquid state [28,29,30] at arbitrarily low pressures and durations of simulation. The lowest width *h* = 5 Å, on the other hand, corresponds to the narrowest pores wettable by water below the experimentally feasible compression limit of ~8 kbar. In simulations on the expulsion branch (initiated from water-filled states), the lateral dimensions of the simulation box, *L*_x_ and *L*_y_, were fixed at 25 Å, whereas we used *L*_y_ at 50 Å in simulations along the intrusion branch, which were generally initiated in a half-full configuration (Figure 2). Depending on the width *h* and bulk pressure *P*_b_, water-filled boxes contained up to ~300 water molecules. For the sake of simplicity, we treat the pores as rigid objects with fixed diameter and volume, neglecting any deformations in principle conceivable [44,45] at high compression. In simulations of the bulk aqueous phase, we used a cubic box of size 25 Å, which contained 15,000 or more molecules. Precise numbers of molecules depended on the pressure, which was controlled through the imposed chemical potential. To establish a connection and enable comparisons with previous works [20,24,25,27,29,30,31,36,46,47,48], we described water molecules using the SPC/E model of water [49], which remains among the standard models for simulation studies of bulk and interfacial water. Interactions between water oxygen atoms (O) and either of laterally extended hydrocarbon-like walls (CH_n_) are approximated by the integrated (9-3) Lennard-Jones (LJ) potential [28,50,51] in the following equation:

(1) $$U_{OCH_{n}}(z)=A{\left(\frac{\sigma_{OCH_{n}}}{\left|z-z_{w}\right|}\right)}^{9}-B{\left(\frac{\sigma_{OCH_{n}}}{\left|z-z_{w}\right|}\right)}^{3}$$

where *z* and *z*_w_ are the z coordinates of the oxygen atom of water and the surface layer of CH_n_ groups on the wall measured from the central (*xy*) plane of the pore [28,30]. $A=\frac{4}{45}\pi \rho {\sigma_{OCH_{n}}}^{3}\varepsilon_{OCH_{n}}$; $B=\frac{15A}{2}$; *ρ* = 0.33 Å^−3^ is the number density of $CH_{n}$ groups in the wall; and $\sigma_{OCH_{n}}$ = 3.454 Å and $\varepsilon_{OCH_{n}}$ = 630 J mol^−1^ are the LJ contact distance and energy parameters determined by Lorentz–Berthelot mixing rules. The selected interaction parameters yield a water–wall contact angle [30] of approximately 127°. In addition to the accelerated computations, the smooth potential from Equation (1) has the advantage of supporting the numerical box-scaling approach [46,52,53] in the finite difference calculation of the lateral component of the pressure tensor in the confinement. While approximate, previous works [30,54,55] manifest essential equivalence of the integrated (9-3) LJ potential (Equation (1)) and atomistic representation in studies of wetting transitions at hydrocarbon walls after appropriate adjustments of $\varepsilon_{OCH_{n}}$ [56] to account for the strengthening of the *averaged* water–wall attraction in the presence of fluctuating topological and temporal patterning [57,58] in the full-atom approach. A systematic comparison between several atomistic models and a related continuum plate model showed no significant advantage of explicit atomistic representation in wall wettability calculations [59]. The confinement is open to the exchange of water molecules in a bulk environment of variable input pressures (*P*). The temperature (*T*) was held at 298 K in all cases.

To consider laterally extended pores, we used periodic boundary conditions along the *x* and *y* directions and calculated their electrostatic interactions using Ewald summation with a two-dimensional correction term introduced by Yeh and Berkowitz [60]. Real-space electrostatic and intermolecular LJ interactions were truncated at a cutoff distance *R*_c_ = 9.8 Å, and we used the screening parameter *α* = π/*R*_c_. Lateral periodicity is implicit in the water/wall interactions described by Equation (1), which relies on the semi-infinite dimensions of the walls. No truncation of oxygen-CH_n_ interactions was used in describing water–wall interactions. The use of tail correction for water/water interactions [30] is precluded by density inhomogeneities [31] associated with filling and evacuation processes in the pores.

### 3.2. Simulation Methods

Simulations of water-filled pores in equilibrium with an implicit bulk phase were performed using the conventional Grand Canonical Monte Carlo [28,46] technique, which determines acceptance probabilities [61] of molecule additions or removals, ${acc}_{N\to N\pm 1}$, according to the following equations [62,63]:

(2) $${\;acc}_{N\to N+1\;}=min\;\left\{\;\frac{<N>}{N+1}e^{\beta \left[\mu_{ex}\left(P_{b}\right)-\Delta U\right]}\;\right\}$$

(3) $${acc}_{N\to N-1}\;=min\;\left\{\;\frac{<N>}{N}e^{\beta \left[{-\mu }_{ex}\left(P_{b}\right)-\Delta U\right]}\right\}$$

where *N* is the number of water molecules in the simulation box, <*N*> is the number of molecules in identical volume in the bulk phase under pressure *P*_b_, $\beta =\frac{1}{kT},\;\mu_{ex}(P_{b})$ is the pressure-dependent excess chemical potential in the bulk aqueous phase, and ΔU denotes the energy change associated with the attempted move. Addition and deletion attempts were combined with translational and rotational molecular moves. Occasional translations of both walls in a perpendicular direction [28,31] proved instrumental in accelerating structural equilibration and convergence of water content in the pore. The acceptance of these moves was determined according to the standard Metropolis algorithm as described in earlier works [28,30]. $\mu_{ex}(P_{b})$ was determined according to the following equation:

(4) $${\;\mu }_{ex}\left(P_{b}\right)=\mu_{ex}\left(P_{o}\right)+\int_{P_{o}}^{P_{b}}\bar{V}\left(P\right)\;dP-kTln\frac{\rho \left(P_{b}\right)}{\rho \left(P_{o}\right)}$$

where *P*_o_ is the ambient pressure (1 bar). At *T* = 298 K and for the SPC/E model of water, ${\beta \mu }_{ex}$(*P*_o_) = −11.88 [31,64], and $\bar{V}$(*P*) = $\bar{V}$($P_{o}$) + $\Delta \bar{V}$(*P*) is the partial molar volume of SPC/E water estimated by using the volumetric data for $\Delta \bar{V}$(*P*) [65] increased by a factor of 1.033 to account for the slightly higher compressibility of SPC/E water [34]. The pressure tensor inside the pores is strongly anisotropic. In hydrophobic pores, the lateral pressure components *P*_||_ = *P*_xx_ = *P*_yy_ are generally smaller than the normal pressure $P_{⊥}=$*P*_zz_. The wetting free energy, defined as the derivative of the system’s grand potential (Ω) with respect to the wetted area of hydrophobic pores, is given by the relation $\Delta \gamma =-\frac{1}{2}h$P_||_ >0. Virial contributions to simulated *P*_||_ and $P_{⊥}$ are calculated using a three-point finite difference approximation to the derivative of configurational energy with respect to uniform box expansion in lateral and normal directions [46,52,53]:

(5) $$\;\;\;\;\;\;\;\;P_{\alpha }=\rho kT+\underset{\Delta V_{\alpha }\to 0}{\lim}\frac{ln<e^{-\frac{\Delta U_{\alpha }}{kT}}>}{\Delta V_{\alpha }}≅\rho kT-\frac{1}{\mathrm{kt}}\underset{\Delta V_{\alpha }\to 0}{\lim}<\frac{\Delta U_{\alpha }}{\Delta V_{\alpha }}>$$

Above, the angular brackets denote the ensemble average; $\alpha =||\;or⊥,\;\Delta U_{\alpha }$ is the energy change associated with minute box scaling along direction α. Most of the GCMC simulations were performed using an in-house Monte Carlo code outlined in ref. [46], augmented by slab-corrected Ewald summation [60] and a routine for chemical potential calculation as a function of compression, Equation (4).

## 4. Concluding Remarks

Reversible wetting/dewetting cycling in a hydrophobic porous medium provides a mechanism for energy storage in the form of interfacial free energy. In water-containing media with a high specific surface area, absorbed energy densities of just below 10^2^ J g^−1^ have been observed in porous-mineral experiments; however, the recovered work can be reduced by pressure- or wetting-induced changes in the absorbent and due to the hysteresis of the intrusion–expulsion cycling. The density of recovered work has so far not exceeded ~30 J g^−1^. Using open ensemble molecular simulations, we examined possible improvements in the cycling efficiency achievable by systematically reducing the width of the absorbent pores. An increased sensitivity of recoverable energy to pore size is observed upon approaching the monolayer width of confined water, thus increasing steric wall–water repulsion and lowering the activation barrier to expulsion. Computed results for cycling hysteresis in subnanometer-model pores of planar geometry show about 25% of stored energy is recovered at 10 Å width and about 92% in twice-narrower pores. In the latter case, water–wall interactions become dominated by packing constraints, irrespective of the nominal hydrophobicity of the wall material. In addition to the near-reversible operation characteristic of liquid springs, molecular-scale porosity optimizes the accessible energy density and can accelerate the kinetics of liquid expulsion, controlling the power density of the system.

## Data Availability

Data are contained within the article.

## References

[1] Grosu Y., Mierzwa M., Eroshenko V.A., Pawlus S., Chorazewski M., Nedelec J.M., Grolier J.P.E. (2017). Mechanical, Thermal, and Electrical Energy Storage in a Single Working Body: Electrification and Thermal Effects upon Pressure-Induced Water Intrusion Extrusion in Nanoporous Solids. ACS Appl. Mater. Interfaces.

[2] Ryzhikov A., Khay I., Nouali H., Daou T.J., Patarin J. (2014). Drastic change of the intrusion-extrusion behavior of electrolyte solutions in pure silica (star)BEA-type zeolite. Phys. Chem. Chem. Phys..

[3] Ryzhikov A., Khay I., Nouali H., Daou T.J., Patarin J. (2016). High pressure intrusion–extrusion of electrolyte solutions in aluminosilicate FAU and BEA-type zeolites. Micropor. Mesopor. Mater..

[4] Han A., Qiao Y. (2007). A volume-memory liquid. Appl. Phys. Lett..

[5] Saada M.A., Rigolet S., Paillaud J.-L., Bats N., Soulard M., Patarin J. (2010). Investigation of the Energetic Performance of Pure Silica ITQ-4 (IFR) Zeolite under High Pressure Water Intrusion. J. Phys. Chem. C.

[6] Le Donne A., Tinti A., Amayuelas E., Kashyap H.K., Camisasca G., Remsing R.C., Roth R., Grosu Y., Meloni S. (2022). Intrusion and extrusion of liquids in highly confining media: Bridging fundamental research to applications. Adv. Phys. X.

[7] Tinti A., Giacomello A., Grosu Y., Casciola C.M. (2017). Intrusion and extrusion of water in hydrophobic nanopores. Proc. Natl. Acad. Sci. USA.

[8] Confalonieri G., Daou T.J., Nouali H., Arletti R., Ryzhikov A. (2020). Energetic Performance of Pure Silica Zeolites under High-Pressure Intrusion of LiCl Aqueous Solutions: An Overview. Molecules.

[9] Fraux G., Coudert F.X., Boutin A., Fuchs A.H. (2017). Forced intrusion of water and aqueous solutions in microporous materials: From fundamental thermodynamics to energy storage devices. Chem. Soc. Rev..

[10] Han A.J., Lu W.Y., Kim T., Chen X., Qiao Y. (2008). Influence of anions on liquid infiltration and defiltration in a zeolite Y. Phys. Rev. E.

[11] Eroshenko V.A., Regis R.C., Soulard M., Patarin J. (2001). Energetics: A new field of applications for hydrophobic zeolites. J. Am. Chem. Soc..

[12] Eroshenko V.A., Regis R.C., Soulard M., Patarin J. (2002). The heterogeneous systems ‘water-hydrophobic zeolites’: New molecular springs. Compt. Rend. Phys..

[13] Soulard M., Patarin J., Eroshenko V.A., Regis R. (2004). Molecular spring or bumper: A new application for hydrophobic zeolitic materials. Studies in Surface Science and Catalysis.

[14] Xu R., Pang W., Yu J., Huo Q., Chen J. (2007). Chemistry of Zeolites and Related Porous Materials Synthesis and Structure.

[15] Confalonieri G., Ryzhikov A., Arletti R., Quartieri S., Vezzalini G., Isaac C., Paillaud J.-L., Nouali H., Daou T.J. (2020). Structural interpretation of the energetic performances of a pure silica LTA-type zeolite. Phys. Chem. Chem. Phys..

[16] Cambiaso S.R.F., Tinti A., Bochicchio D., Grosu Y., Rossi G., Giacomello A. (2023). Grafting heterogeneities rule water intrusion and extrusion in nanopores. arXiv.

[17] Grosu Y., Li M., Peng Y.L., Luo D., Li D., Faik A., Nedelec J.M., Grolier J.P. (2016). A Highly Stable Nonhysteretic {Cu-2(tebpz) MOF plus water} Molecular Spring. Chem. Phys. Chem..

[18] Leung K., Luzar A. (2000). Dynamics of capillary evaporation. II. Free energy barriers. J. Chem. Phys..

[19] Leung K., Luzar A., Bratko D. (2003). Dynamics of capillary drying in water. Phys. Rev. Lett..

[20] Sharma S., Debenedetti P.G. (2012). Free Energy Barriers to Evaporation of Water in Hydrophobic Confinement. J. Phys. Chem. B.

[21] Gao Y., Li M.Z., Zhang Y., Lu W.Y., Xu B.X. (2020). Spontaneous outflow efficiency of confined liquid in hydrophobic nanopores. Proc. Natl. Acad. Sci. USA.

[22] Yaminsky V.V., Ohnishi S., Ninham B., Nalwa H.S. (2001). Long-range hydrophobic forces due to capillary bridging. Handbook of Surfaces and Interfaces of Materials.

[23] Luzar A. (2004). Activation barrier scaling for the spontaneous evaporation of confined water. J. Phys. Chem. B.

[24] Ghasemi M., Ramsheh S.M., Sharma S. (2018). Quantitative Assessment of Thermodynamic Theory in Elucidating the Behavior of Water under Hydrophobic Confinement. J. Phys. Chem. B.

[25] Tinti A., Giacomello A., Meloni S., Casciola C.M. (2023). Classical nucleation of vapor between hydrophobic plates. J. Chem. Phys..

[26] Guillemot L., Biben T., Galarneau A., Vigier G., Charlaix E. (2012). Activated drying in hydrophobic nanopores and the line tension of water. Proc. Natl. Acad. Sci. USA.

[27] Bey R., Coasne B., Picard C. (2020). Probing the concept of line tension down to the nanoscale. J. Chem. Phys..

[28] Bratko D., Curtis R.A., Blanch H.W., Prausnitz J.M. (2001). Interaction between hydrophobic surfaces with metastable intervening liquid. J. Chem. Phys..

[29] Giovambattista N., Rossky P.J., Debenedetti P.G. (2006). Effect of pressure on the phase behavior and structure of water confined between nanoscale hydrophobic and hydrophilic plates. Phys. Rev. E.

[30] Moucka F., Bratko D., Luzar A. (2015). Electrolyte pore/solution partitioning by expanded grand canonical ensemble Monte Carlo simulation. J. Chem. Phys..

[31] Zamfir S.G.M.F., Bratko D. (2020). High-Pressure Infiltration–Expulsion of Aqueous NaCl in Planar Hydrophobic Nanopores. J. Phys. Chem. C.

[32] Ronchi L., Ryzhikov A., Nouali H., Daou T.J., Patarin J. (2017). Energetic performances of FER-type zeolite in the presence of electrolyte solutions under high pressure. Energy.

[33] Michelin-Jamois M., Picard C., Vigier G., Charlaix E. (2015). Giant Osmotic Pressure in the Forced Wetting of Hydrophobic Nanopores. Phys. Rev. Lett..

[34] Teplukhin A.V. (2019). Thermodynamic and Structural Characteristics of SPC/E Water at 290 K under High Pressure. J. Struct. Chem..

[35] Lum K., Chandler D., Weeks J.D. (1999). Hydrophobicity at small and large length scales. J. Phys. Chem. B.

[36] Paulo G., Gubbiotti A., Giacomello A. (2023). An atomistically informed multiscale approach to the intrusion and extrusion of water in hydrophobic nanopores. J. Chem. Phys..

[37] Liu J.C., Monson P.A. (2005). Does water condense in carbon pores?. Langmuir.

[38] Tinti A., Camisasca G., Giacomello A. (2021). Structure and dynamics of water confined in cylindrical nanopores with varying hydrophobicity. Phil. Trans. Roy. Soc. A.

[39] Deroche I., Daou T.J., Picard C., Coasne B. (2019). Reminiscent capillarity in subnanopores. Nat. Commun..

[40] Ronchi L., Ryzhikov A., Nouali H., Daou T.J., Patarin J. (2018). Energetic Performances of Pure-Silica DDR Zeolite by High-Pressure Intrusion-Extrusion of Electrolyte Aqueous Solutions: A Shock-Absorber with Huge Absorbed Energy. J. Phys. Chem. C.

[41] Li R.Y.Z., Xia G. (2023). Effect of inter-pore interference on liquid evaporation rates from nanopores by direct simulation Monte Carlo. Phys. Fluids.

[42] Vanzo D., Luzar A., Bratko D. (2022). Pressure-sensitive conversions between Cassie and Wenzel wetting states on a nanocorrugated surface. Appl. Phys. A.

[43] Mehrani R., Sharma S. (2022). Stability of Water Confined between Supported Self-Assembled Monolayers. J. Phys. Chem. B.

[44] Altabet Y.E., Debenedetti P.G. (2014). The role of material flexibility on the drying transition of water between hydrophobic objects: A thermodynamic analysis. J. Chem. Phys..

[45] Altabet Y.E., Haji-Akbari A., Debenedetti P.G. (2017). Effect of material flexibility on the thermodynamics and kinetics of hydrophobically induced evaporation of water. Proc. Natl. Acad. Sci. USA.

[46] Bratko D., Daub C.D., Leung K., Luzar A. (2007). Effect of field direction on electrowetting in a nanopore. J. Am. Chem. Soc..

[47] Ritchie J.A., Yazdi J.S., Bratko D., Luzar A. (2012). Metastable Sessile Nanodroplets on Nanopatterned Surfaces. J. Phys. Chem. C.

[48] Daub C.D., Wang J., Kudesia S., Bratko D., Luzar A. (2010). The influence of molecular-scale roughness on the surface spreading of an aqueous nanodrop. Faraday Discuss..

[49] Berendsen H.J.C., Grigera J.R., Straatsma T.P. (1987). The Missing Term In Effective Pair Potentials. J. Phys. Chem. B.

[50] Lee S.H., Rossky P.J. (1994). A Comparison of the Structure and Dynamics of Liquid Water at Hydrophobic and Hydrophilic Surfaces—A Molecular-Dynamics Simulation Study. J. Chem. Phys..

[51] Shelley J.C., Patey G.N. (1996). Boundary condition effects in simulations of water confined between planar walls. Mol. Phys..

[52] Bratko D., Jonsson B., Wennerstrom H. (1986). Electrical Double-Layer Interactions with Image Charges. Chem. Phys. Lett..

[53] Gloor G.J., Jackson G., Blas F.J., de Miguel E. (2005). Test-area simulation method for the direct determination of the interfacial tension of systems with continuous or discontinuous potentials. J. Chem. Phys..

[54] Moucka F., Bratko D., Luzar A. (2015). Salt and Water Uptake in Nanoconfinement under Applied Electric Field: An Open Ensemble Monte Carlo Study. J. Phys. Chem. C.

[55] Moucka F., Zamfir S., Bratko D., Luzar A. (2019). Molecular polarizability in open ensemble simulations of aqueous nanoconfinements under electric field. J. Chem. Phys..

[56] Vanzo D., Bratko D., Luzar A. (2012). Wettability of pristine and alkyl-functionalized graphane. J. Chem. Phys..

[57] Bratko D., Chakraborty A.K., Shakhnovich E.I. (1996). Frozen phases of random heteropolymers in disordered media. Phys. Rev. Lett..

[58] Bratko D., Striolo A., Wu J.Z., Blanch H.W., Prausnitz J.M. (2002). Orientation-averaged pair potentials between dipolar proteins or colloids. J. Phys. Chem. B.

[59] Jaffe R.L., Gonnet P., Werder T., Walther J.H., Koumoutsakos P. (2004). Water-carbon interactions—2: Calibration of potentials using contact angle data for different interaction models. Mol. Simul..

[60] Yeh I.C., Berkowitz M.L. (1999). Ewald summation for systems with slab geometry. J. Chem. Phys..

[61] Frenkel D., Smit B. (2002). Understanding Molecular Simulation, from Algorithms to Applications.

[62] Adams D.J. (1974). Chemical potential of hard-sphere fluid by Monte Carlo methods. Mol. Phys..

[63] Adams D.J. (1975). Grand Canonical Monte Carlo for Lennard Jones fluid. Mol. Phys..

[64] Moucka F., Nezbeda I., Smith W.R. (2015). Chemical Potentials, Activity Coefficients, and Solubility in Aqueous NaCl Solutions: Prediction by Polarizable Force Fields. J. Chem. Theory Comput..

[65] Adams L.H. (1931). Equilibrium in binary systems under pressure. I. An experimental and thermodynamic investigation of the system, NaC1-H2O, at 25°. J. Am. Chem. Soc..

